# Potential markers of cancer stem-like cells in ESCC: a review of the current knowledge

**DOI:** 10.3389/fonc.2023.1324819

**Published:** 2024-01-04

**Authors:** Lu Wang, Huijuan Liu, Yiqian Liu, Shixing Guo, Zhenpeng Yan, Guohui Chen, Qinglu Wu, Songrui Xu, Qichao Zhou, Lili Liu, Meilan Peng, Xiaolong Cheng, Ting Yan

**Affiliations:** ^1^ Translational Medicine Research Center, Shanxi Medical University, Taiyuan, Shanxi, China; ^2^ Key Laboratory of Cellular Physiology of the Ministry of Education, Department of Pathology, Shanxi Medical University, Taiyuan, Shanxi, China; ^3^ Clinical Laboratory Medicine Centre, Shenzhen Hospital, Southern Medical University, Shenzhen, China

**Keywords:** esophageal squamous cell carcinoma, cancer stem-like cells, EMT, surface markers, microenvironment

## Abstract

In patients with esophageal squamous cell carcinoma (ESCC), the incidence and mortality rate of ESCC in our country are also higher than those in the rest of the world. Despite advances in the treatment department method, patient survival rates have not obviously improved, which often leads to treatment obstruction and cancer repeat. ESCC has special cells called cancer stem-like cells (CSLCs) with self-renewal and differentiation ability, which reflect the development process and prognosis of cancer. In this review, we evaluated CSLCs, which are identified from the expression of cell surface markers in ESCC. By inciting EMTs to participate in tumor migration and invasion, stem cells promote tumor redifferentiation. Some factors can inhibit the migration and invasion of ESCC via the EMT-related pathway. We here summarize the research progress on the surface markers of CSLCs, EMT pathway, and the microenvironment in the process of tumor growth. Thus, these data may be more valuable for clinical applications.

## Introduction

In the past year, esophageal cancer (EC) has increased by 572,000 new cases and 509,000 deaths globally. It ranks seventh in occurrence in all kinds of cancers, which means one of 20 cancer deaths was due to EC (1). Tumor mass is a heterogeneous hierarchy. Most of the cells could no longer differentiate. Only a small set of them has the capacity of self-renewal and could differentiate into malignant cancer cells (2). This small group of cells is called CSLCs, or tumor-initiating cells (TICs). Theories of CSLCs believe that the poor effect of cancer therapies, which showed relapse and metastasis of cancer cells, might be because of the therapeutic resistance of CSLCs (3, 4). For example, in breast cancer cells, studies show that miR-155 enhances stemness, decitabine (DCA) resistance, and CSLC properties by targeting TSPAN5, which causes TNBC to have an unfortunate forecast (5). Studies by Li et al. showed that single-cell RNA sequencing in hepatocellular carcinoma has produced an abundance of information to validate a panel of cells with cancer stem-like cells’ properties (6). In cancer treatment, only the differentiated cells could make a response. However, the surviving CSLCs may differentiate into new cancer cells. CSLCs are thought to be seed cells in the process of tumor formation, control of the occurrence, and metastasis through complex signal transduction (7). In light of this feature, it should be better to target the CSLCs in cancer treatments. The targeted molecules specific to CSLCs became a hot topic in cancer research.

Up to now, a good deal of studies have shown the existence of CSLCs in ESCC, and their functions involve proliferation and tumor growth and even indicate poor prognosis. In this review, that is the reason why we explain recent advances in identification markers of CSLCs and the link between CSLCs and EMT and the immune cell microenvironment. There is a need to create new therapies for CSLCs in ESCC.

## Materials and methods

We followed the PRISMA 2020 rules and applied for our review (8). The articles were carefully reviewed using literature resources such as PubMed service of the US National Library of Medicine and Geen Medical. Search algorithms such as “ESCC”, “EMT”, “cancer stem-like cells”, “marker”, “cancer”, “tumor”, and “pathway” were used in searches. In this review, references to retrieved articles were also filtered for additional data. It is important to note that the studies described in this article did not use any data (**Supplementary Figure S1**).

### Overview of CSLC marker in ESCC

**Figure 1:**
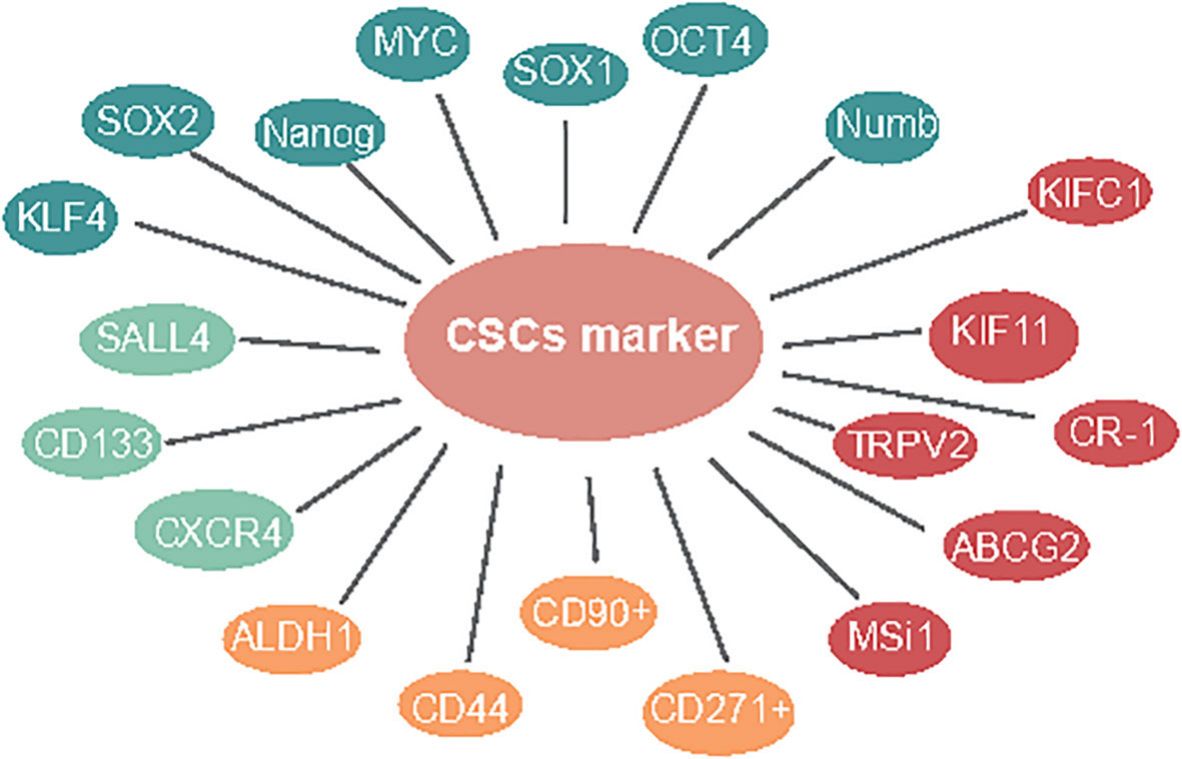
Markers in ESCC CSLCs.

Studies suggest that focusing on CSLC marker-based treatments might act as a more powerful procedure to take out these recalcitrant cells (9). These markers and some signaling pathways may also serve as targets for the elimination of CSLCs (10). First, Mardani et al. showed that co-articulation of CSLC markers CD133/CXCR4 might have a poor prognosis in osteosarcoma. Meanwhile, CD133/SALL4 has a critical relationship among SALL4 and BMP signal target genes, including SIZN1, VENTX, and DIDO1. It assumes a significant role in tumorigenesis in ESCC (11). Next, for CSLC therapy, SN-38 is a nanocarrier for topoisomerase inhibitors; CD133 is a theoretical CSLC marker; CD133-NPS-SN-38 represses growth development and can dispose CD133-positive cells, which is a potential CSLC-designated treatment (12). Therefore, the exploration of more ESCC-CSLC markers on the surface can provide a basis for the recognizable proof of CSLCs and targeted therapy of CSLCs (**Figure 1**, green).

Similar to other kinds of solid tumors, several cell surface molecules have been recognized as markers of ESCC-CSLCs. Wang and Yang exhibited that ALDH1-expressing cells are highly invasive metastases in ESCC (13, 14). ALDH1 is accounted for as a marker of normal and malignant stem cells in several lineages. Nuclear expression of ALDH1 is related to lymph hub metastasis and low endurance in ESCC (13–15). Based on this research, the expression of ALDH1 was associated with a poor prognosis in 577 cases of breast cancer (16). CD44 has been utilized as a cell surface marker for stemness, has CD133. It was also confirmed as the CSLC marker in ESCC cell lines (17) that could be utilized to efficiently enrich TICs (17). One study showed cells with CD44^High^/CD24^Low^, which is a recognized marker for CSLCs in breast cancer (18), have been confirmed to possess CSLC properties (19). Additionally, CD90+ cells show an improved capacity for self-renewal, differentiation, and resistance to chemotherapy (20). CD271^+^ malignant growth cells showed higher sphericity and state-framing limits, high articulation of immature microorganism-related qualities, and protection from chemotherapy (21) (**Figure 1**, yellow).

CSLC surface markers are important for targeted therapy in ESCC. Similarly, we also believe that regulating genes associated with stem cell markers is important. The inhibition of TRPV2 by low concentrations of Tranilast is more cytotoxic in CSLCs than in the non-CSLC population, indicating that Tranilast could be utilized as a novel targeted therapeutic agent against ESCC-CSLCs (22). ABCG2 (23) and Msi1 (24) overexpression cells were found to represent CSLCs with special harmful potential in ESCC and could regulate the proliferation, apoptosis, sphere formation, and migration ability in spheroid cells (25). Cripto-1-positive ESCC cells were higher stemness-related genes, self-renewal, tumorigenesis, boosting tumor cell migration, invasion, and angiogenesis (26). Moreover, it has been reported that KIFC1 (27) and kinesin family 11 (KIF11) (28) were overexpressed and required for sphere formation in ESCC cells. Interestingly, in cells with Ras-like expression without CAAX1 (RIT1) exogenously overexpressing, the stemness genes, for example, ALDH1, ABCG2, OCT4, CD44, and CXCR4, were significantly downregulated (29) (**Figure 1**, red).

The green illustrates CD133, CXCR4, and SALL4 presented in a one-paragraph overview in CSLC marker in ESCC. The yellow illustrates ALDH1, CD44, CD90^+^, and CD271^+^ presented in a two-paragraph overview of CSLC markers in ESCC. The red illustrates TRPV2, ABCG2, MSi1, CR-1, KIFC1, and KIF11 presented in a three-paragraph overview of CSLC markers in ESCC. The blue illustrates OCT4, SOX1, MYC, Nanog, KLF4, SOX2, and Numb, presented in a four-paragraph overview of CSLC markers in ESCC.

The organic action of CSLCs is controlled by pluripotent record factors like SOX2, MYC, KLF4, OCT4, and Nanog (30). Moreover, studies have shown that pluripotent stem cells could be produced straightforwardly from the fibroblast culture with certain factors, such as Oct3/4, c-MYC, and Sox2 (31). The immature microorganism marker Nanog regulates stem cell differentiation, proliferation, and asymmetric division (32). Du et al. demonstrated the overexpression of Nanog and that a mix of Nanog siRNA with cisplatin showed further improved chemosensitivity in ESCC (33). While SALL4 is obviously increased in cell spheres, which is deemed as an enrichment of CSC-like cells (34), SOX1, a tumor-suppressor gene, was shown to be underexpressed combined with SALL4 overexpression in ESCC and showed a critical role in the inhibition of aggressiveness, indicating the therapeutic potential of the molecule against ESCC-CSLCs (35). Furthermore, the downregulation of the Numb inhibited cell proliferation and expression of CSLC markers (36) (**Figure 1**, blue).

### EMT pathway and tumor microenvironment in ESCC-CSLC targeting

In addition to marker identifications, studies started to focus on exploring CSC features, such as the tumorigenesis, metastasis, and therapeutic resistance role of CSLCs in ESCC. However, when the tumor metastases, the primary tumor needs to invade the blood vessels, and the distant metastasis needs to activate EMT to dedifferentiate so that the cancer cells can spread and move (37) so that the cancer stem-like cells can migrate and move (38). In addition, cancer-associated EMT results in more migratory cells capable of forming new tumor tissue, indicating increased stemness (38, 39). In that way, whether EMT triggers tumor progression by stimulating CSLC’s potential. One study identified that Twist1 is an important transcription factor that upregulates the expression of Oct4 protein and Sox2 protein (40, 41). Knocking down USP4 resulted in a decrease in OCT4 and SOX2 proteins (42). Evolving evidence suggests that CTAs induce EMT and CSLC generation (43). In addition, silencing SRPX2 inhibited cell proliferation and EMT via the Wnt/β-catenin pathway, increasing sensitivity toward cisplatin for ESCC cells (44). Therefore, this article means to sum up the progress of EMT regulatory mechanisms, aiming to elucidate the potential role of EMT in CSLC-targeted therapy and tumor resistance research.

### EMT-associated pathway in CSLCs

EMT-related signaling pathways are involved in the survival, self-renewal, and differentiation of ESCC-CSLCs. These signaling pathways form a network of interlaced signaling media that regulate the growth of CSLCs. The investigation of small-molecule inhibitors of this signal pathway is a functioning area of cancer drug advancement (45). It has been found that the abnormal activation of Notch, NF-κB, and Wnt signaling pathways can result in the proliferation, differentiation, and self-renewal of CSLCs. It is suggested that Notch can induce the growth of CSLCs and maintain the stem cells, which is an effective measure to treat tumors and reverse the drug resistance of tumor chemotherapy. Therefore, the following describes how signaling pathways are regulated.

### TGF-β signaling pathway in ESCC (ESCC)

**Figure 2:**
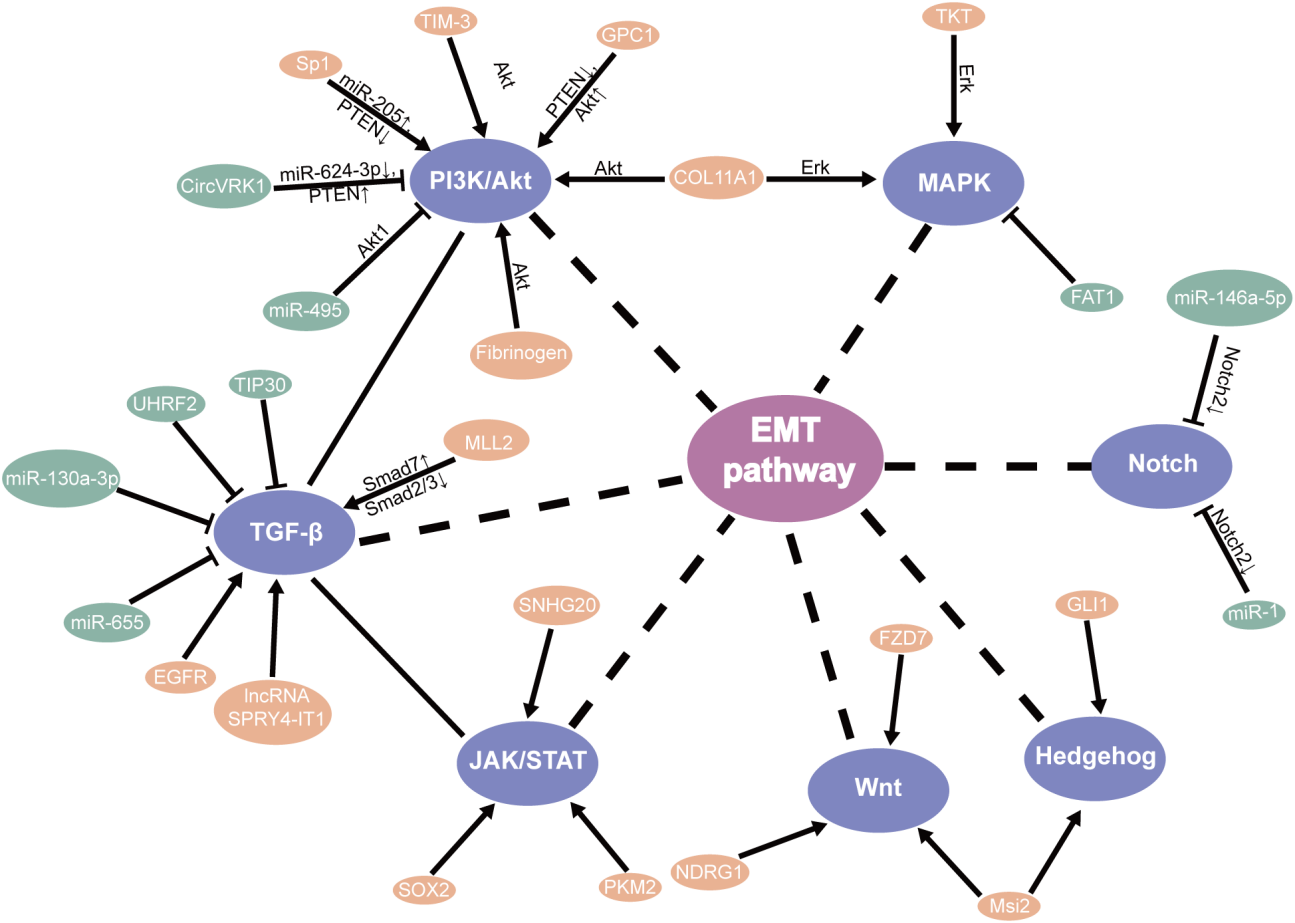
EMT-related signaling pathways in ESCC CSLCs.

TGF-β signaling plays a protumor role by promoting EMT, migration, and invasion (46). Relevant studies have shown that knockdown TIP30 can result in EMT via the TGF-B pathway, improving the invasive ability and advancing growth metastasis in *in vivo* and *in vitro* (47). Additionally, MLL2 (48), EGFR (49), and LncRNA SPRY4-IT1 (50) were found to directly promote EMT through the TGF-β pathway in ESCC, which may participate EMT for patients with ESCC through the different mechanisms (51). Meanwhile, UHRF2 and miR-655 were recognized to suppress EMT by the TGF-β pathway (52, 53). Based on research, Tian et al. found that miR-130a-3p suppressed TGF-β-promoted EMT progression in a SMAD4-dependent manner in ESCC (54). Moreover, the PTEN/PI3K/Akt signaling pathway is the major regulator that promotes EMT progress in ESCC. Hence, these should be considered in the strategies against EMT (55–57) (**Figure 2**).

### PI3K/AKT signaling pathway in ESCC and association with its targeting CSLCs

The PI3K/AKT Signaling pathway has been demonstrated to be essential to the regulator of CSLCs by EMT (30). AKT is a vital individual from the PI3K/AKT signal pathway, which has been shown to promote the progression of multiple cancers, especially in the self-renewal of CSLCs (58).

**Figure 3:**
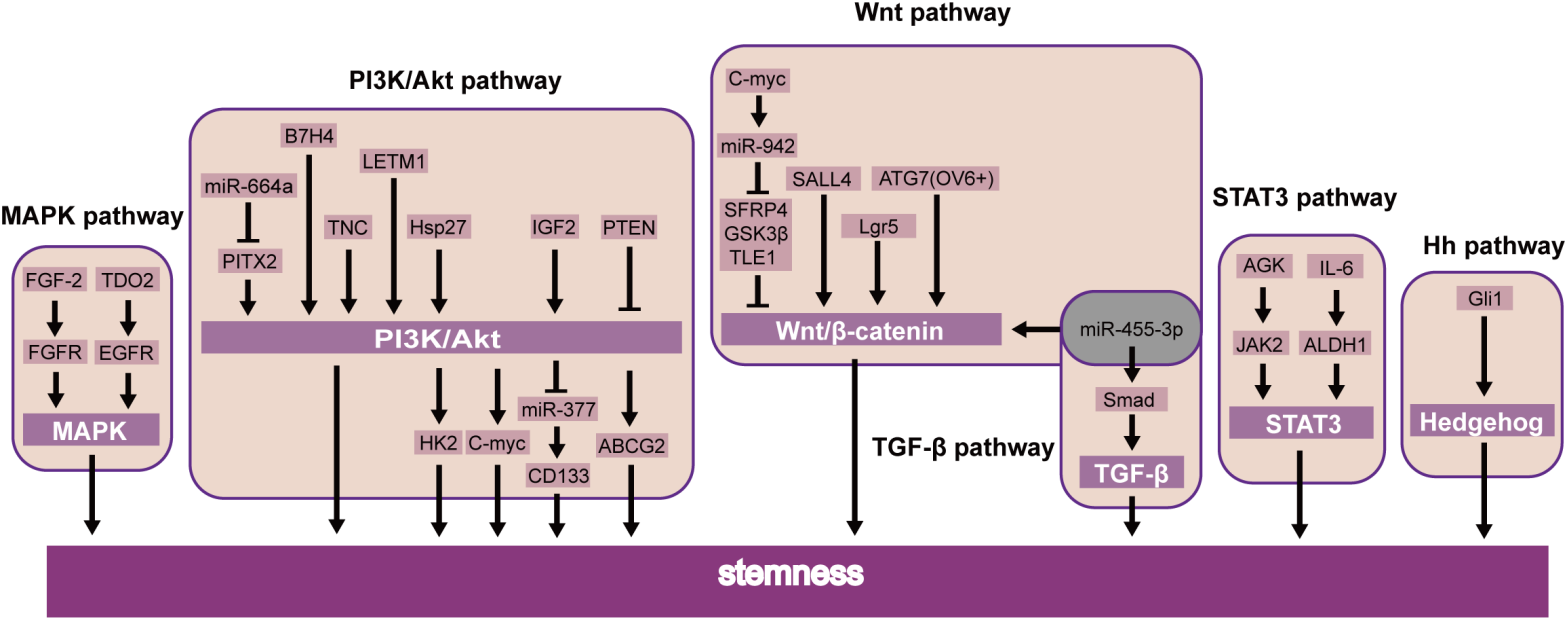
Activated and inhibitor pathways in ESCC CSLCs.

Recent studies showed that B7H4 (59), TNC (60), and LETM1 (61) were further confirmed to induce CSLC character through the PI3K/AKT pathway. Additionally, miR-664a attenuates stem-cell-associated phenotype and ESCC cell malignancy, in part due to the inactivation of the Akt/GSK-3β/β-catenin pathway through Pitx2 (62). Li et al. also showed that PTEN was also involved in the PI3K/Akt/ABCG2 pathway and regulated the CSLC population of ESCC (63). Further studies have shown that stem cell properties of drug resistance, tumor initiation, an increase of glycolysis, and oxidative phosphorylation are dependent on the Hsp27-AKT-HK2 pathway in ESCC (64). Nevertheless, it upholds the significance of the IGF2-PI3K/AKT-miR-377-CD133 axis in maintaining the malignant growth of CSLCs (65). Interestingly, CD133 has been found downstream of PI3K/AKT/miR-377 to mediate the functions of CSLCs (65). Simultaneously, the PI3K-AKT signaling pathway can upregulate c-MYC, which will promote stemness in ESCC (66) (**Figure 3**).

In addition, the PI3K/mTOR signal pathway plays a significant part in cell proliferation and survival. Studies have shown that miR-495 suppresses proliferation, migration, and invasion in ESCC cells by AKT1 (67). It has been reported and demonstrated that circVRK1 suppressed EMT progression and radioresistance. The possible worth of circVRK1 on ESCC was proposed by miR-624-3p/PTEN and the PI3K/AKT signal pathway (68). Conversely, knockdown of TIM-3 suppressed EMT through the Akt/GSK-3β/Snail pathway in ESCC (69). It was likewise recommended that fibrinogen promoted EMT via the p-AKT/p-mTOR pathway to increase cell motility (70). One study showed that silencing Rab3D inhibited the proliferation by the PI3K/Akt pathway in ESCC (71). Furthermore, Glypican-1 (GPC1), via the regulation of the PTEN/Akt/β-catenin signaling pathways, and Sp1/miR-205 via the PTEN/PI3K/Akt pathway (72) directly enhances EMT in ESCC (73). These discoveries suggested that they might be a new therapeutic target and prognostic biomarker for ESCC through the PI3k/Akt pathway (**Figure 2**).

### JAK-STAT signaling pathway in ESCC (ESCC) and association with its targeting CSLCs

Moreover, transcription factors likewise prompt the self-renewal of CSLCs via the JAK-STAT signaling pathway. STAT3 is essential for self-renewal in embryonic stem cells (74). Current studies have focused on its role in oncogenesis. In breast cancer, STAT3 induces cell proliferation and maintains CSLC stemness (75). Similarly, the same effect was demonstrated by another group by means of the AGK/JAK2/STAT3 axis. Patients with ESCC had a more limited general endurance and a more terrible sickness-free endurance (76). Interestingly, STAT3β inhibited chemoresistance and stemness through STAT3α (77), which requires further clinical investigations (**Figure 3**).

The impact of other transcription factors’ expression was induced/reduced by cell migration, invasion, and EMT by the JAK-STAT pathway. Gao et al. also concluded that it was associated with SOX2-incited Slug-interceded EMT (78). Moreover, PKM2 promoted the progress of EMT which induced TGF-β1 via phosphorylation STAT3 (79). Furthermore, SNHG20 affects EMT by ATM/JAK/PD-L1 pathway in ESCC (80) (**Figure 2**).

### MAPK signaling pathway in ESCC and association with its targeting CSLCs

The MAPK pathway responds to multiple input signals as growth factors (75). Research has demonstrated that the MAPK signaling pathway is a valid target for cancer treatment. For example, the best progress has been made in drug targets by the RAS-RAF-MEK-ERK axis (81). Furthermore, some findings indicate that the FGF-2/FGFR (19) and TDO2/EGFR (82) axes were essential factors regulating CSLCs via the MAPK pathway in ESCC. These transcription factors could be potential targets for ESCC through MAPK stemness (**Figure 3**).

In addition, other factors were shown to modulate the EMT through the MAPK signaling pathway. Our early research suggested inhibition of FAT1 promotes the progression of EMT, which induces the MAPK/ERK pathway in ESCC (83). Meanwhile, relevant research showed that triptolide suppresses cell proliferation, invasion, and migration through the MAPK/ERK pathway in ESCC (84). For example, TKT has been identified as a critical determinant that promotes cell invasion by mediating the EMT process, leading to esophageal cancer (85). Knockdown of COL11A1 inhibited migration and invasion capabilities by EMT (86) (**Figure 3**).

### Wnt, Hh, and Notch signaling pathways in ESCC and their association with their targeting CSLCs

The Wnt pathway may directly regulate the self-renewal of CSLCs (46). MiR-455-3p can promote chemoresistance and tumorigenesis in ESCC cells through the Wnt/β-catenin pathway and the TGF-β/Smad pathway (87). Taken together, SALL4 (34), Lgr5 (88), and ATG7 (89) can also regulate CSLC proliferation by the Wnt/β-catenin pathway in ESCC. c-Myc combined the miR-942 promoter and suppressed sFRP4, GSK3β, and TLE1, which regulated the Wnt/β-catenin pathway (90). A similar study of CSLCs of patients with esophageal cancer also showed the Hedgehog pathway, a key signaling for stemness maintenance of ESCC cells, played a role in the self-renewal of ESCC-CSLCs based on overexpression of glioma-associated oncogene homolog1 (Gli1) (91). The current study found that the Wnt inhibitor IWP-2 can target the Wnt pathway. Therefore, by inhibiting the Wnt signaling pathway, it can inhibit the growth of CSLC and achieve the goal of treating cancer (92). The above experimental results suggest that these factors may be used as new prognostic biomarkers or therapeutic targets in ESCC (**Figure 3**).

Aberrant Notch signaling promotes self-renewal and the transfer of mammary stem cells (93). Stearoyl-coa desaturase-1 (SCD1) has been found to be important in the survival of CSLCs. SCD1 inhibitors can significantly reduce the Notch signaling pathway, which further damages CSLC and increases the sensitivity of tumors. Therefore, SCD1 may be a new target in colorectal cancer (94). The Hedgehog pathway assumes a significant part in cancer through EMT (95). Strikingly, increased levels of N-myc-downregulated NDRG1 activated the Wnt pathway and EMT, which decreased the expression of TLE2 and increased β-catenin in ESCC (93). Cao et al. also concluded that FZD7 promoted the progress of EMT through the Wnt/β-catenin pathway in ESCC (96). Additionally, the presence of Msi2 promotes ESCC cell proliferation, between Hedgehog (Hh) and Wnt/β-catenin by EMT pathways (97). Furthermore, the EMT regulator SIP1 is positively regulated by the Hh signal sensor GLI1 (95). On the other hand, Notch2 as the target gene for miR-146a-5p and miR-1 (98) inhibits EMT in ESCC (50). In particular, knockdown of NHE leads to EMT by inhibiting the Notch3 pathway in ESCC (99) (**Figure 2**).

### The microenvironment associated with EMT in CSLCs

**Figure 4:**
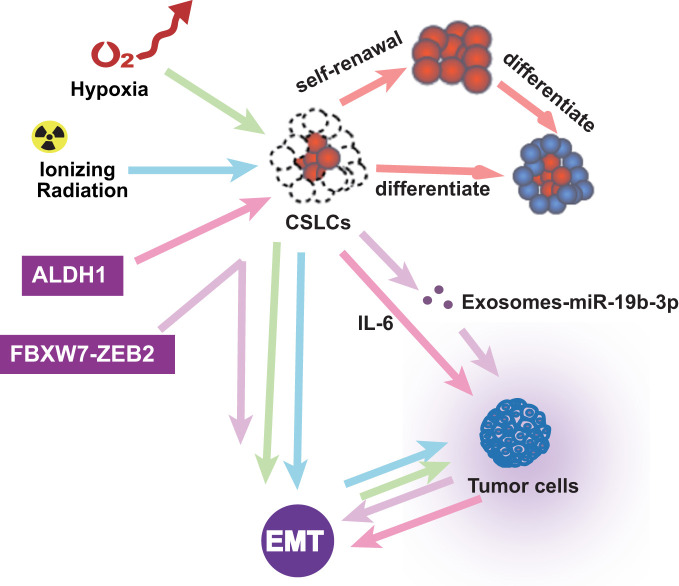
The microenvironment associated with EMT in ESCC CSLCs.

In addition to pathway and transcription factors, EMT-related microenvironments are also noteworthy. CSLCs were regarded as super stem cells and out of control (100). In the tumor microenvironment, inflammatory cells and molecules influence almost every process. Research showed that FBXW7-ZEB2 regulates the drug resistance and migration of tumor cells (101). Chronic tumor-associated inflammation is a marker that stimulates the progression of metastasis in cancer (5). The tumor microenvironment is also essential in EMT. Ionizing radiation is known to induce the self-renewal of CSLCs and promote tumor progression by activating EMT (102). Hypoxia induces EMT, in which only the cancer stem-like cells induce invasion and metastasis (103). CSLC exosomes transported by miR-19b-3p promote cell proliferation and active EMT (104). ALDH1-positive tumors are associated with aggressive tumor growth through EMT and IL-6 increases (105). The cancer microenvironment assumes a significant part in prompting EMTs and keeping up with CSLCs. These studies reveal interactions between different types of cells in the tumor microenvironment and their impact on promoting EMT and enhancing the self-renewal of CSLC. Therefore, there is a bidirectional relationship among tumor microenvironment, EMT, and CSLC, which affect each other and promote the development of tumors and the formation of drug resistance (**Figure 4**).

## Discussion

CSLCs are believed to be the main cause of the development of most solid tumors and the major factor in drug resistance. Targeting cancer patients with stem cells is promising for the future. Therefore, effective molecular targets for CSLCs must be carefully selected, and the mechanism of the targeted therapy for CSLCs must be thoroughly revealed. This paper reviews the characteristics and identification of CSLCs and discusses the potential targeted therapies for CSLCs. The identification of specific early diagnosis and prognostic CSLC markers in ESCC gives a strategy for the classification of diseases. In order to improve the outcomes of ESCC treatment, new targeted CSLC therapies are also needed. The above provides insights into how ESCC CSLCs initiate cancer and treat resistance and metastasis. At present, drug development for cell signaling has become a new type of chemotherapy. It is worth considering that, despite the focus on key signaling pathways and their potential as a potential treatment strategy, the trial failure rate remains high. Many drugs may work, but they may have different effects for patients at different times. This highlights the importance of precision treatment. The different roles of CSLCs in ESCC emphasize the importance of their related genes as therapeutic targets.

Advances in translational medicine have enabled us to better understand the role and outcomes of cancer therapies. In particular, the introduction of the CSC concept and the link between EMT and CSC provide us with new insights into solving ESCC problems. In addition, EMT may be the main pathway for ESCC cells to obtain the CSC phenotype, which makes it a powerful new target for ESCC treatment. That makes these pathways attractive targets for cancer treatment. Therefore, further study is needed on the association between EMT and CSC in order to use the EMT-CSC link to improve treatment practices.

However, there are still many obstacles to the complete elimination of stem cells. First, stem cells have not yet been accurately identified. Second, some of the current ESCC-CSC studies are in bulk cell research. Due to the limitations of research methods, it is difficult to study the function of related genes in SP. Cell experiments and other *in vitro* experiments cannot fully reflect the changes in the human body. In the context of precision medicine, patients derived from ESCC organoids can serve as a reliable model system for studying tumor evolution and treatment response. Therefore, the development of new methods is very important. Third, in light of the fact that CSLCs additionally share some pathways with normal cells, not all controllers that cause CSLCs are appropriate as focuses for disease treatment. Fourth, we need to pay more attention to the role of natural products targeting CSLCs in research. For example, curcumin cannot only clear cancer cells but also target tumor cells (106). Fifth, related signaling molecules have emerged as potential stem cell therapies. Therefore, multitarget inhibitors will be one of the fundamental techniques to conquer the drug resistance of CSLCs (106). Sixth, CSLC therapy targets the activation or inhibition of stem cells to promote or prevent CSLCs from entering the cell cycle, which is also a problem worth considering (107). Finally, the treatment of cancer with CSLCs as the target is very promising, which is a hot topic at present and needs to be further explored.

## Author contributions

LW: Investigation, Writing – original draft. HL: Data curation, Supervision, Writing – review & editing. YL: Methodology, Supervision, Writing – review & editing. SG: Investigation, Writing – review & editing. ZY: Methodology, Conceptualization, Data curation, Writing – review & editing. GC: Formal analysis, Writing – review & editing. QW: Formal analysis, Writing – review & editing. SX: Formal analysis, Writing – review & editing. QZ: Writing – review & editing, Investigation, Software. LL: Methodology, Writing – review & editing. MP: Methodology, Writing – review & editing. XC: Writing – review & editing, Methodology, Formal analysis, Validation. TY: Conceptualization, Funding acquisition, Writing – review & editing.
