# Supplementary material for: Potential markers of cancer stem-like cells in ESCC: a review of the current knowledge
Source: Front Oncol. 2024 Jan 4;13:1324819. doi: 10.3389/fonc.2023.1324819 (PMC10795532; doi:10.3389/fonc.2023.1324819)
Supplement: Supplementary file 1 [file DataSheet_1.pdf]

PRISMA 2020 flow diagram for new systematic reviews which included searches of databases and registers only

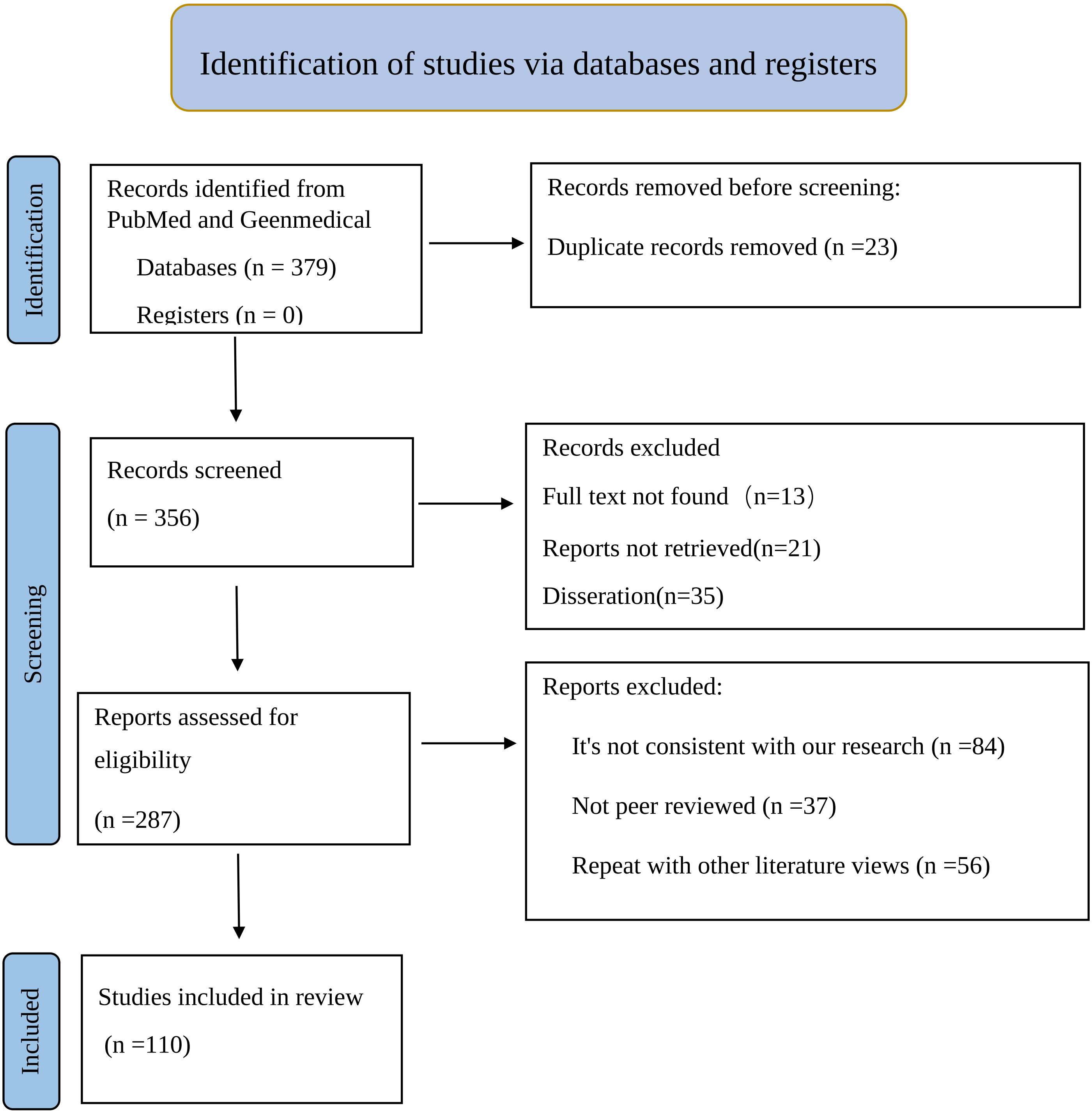

*Consider, if feasible to do so, reporting the number of records identified from each database or register searched (rather than the total number across all databases/registers).

**If automation tools were used, indicate how many records were excluded by a human and how many were excluded by automation tools.

From: Page MJ, McKenzie JE, Bossuyt PM, Boutron I, Hoffmann TC, Mulrow CD, et al. The PRISMA 2020 statement: an updated guideline for reporting systematic reviews. BMJ 2021;372:n71. doi: 10.1136/bmj.n71

For more information, visit: http://www.prisma-statement.org/
